# Collaborative research between clinicians and researchers: a multiple case study of implementation

**DOI:** 10.1186/1748-5908-5-76

**Published:** 2010-10-14

**Authors:** Dean Blevins, Mary S Farmer, Carrie Edlund, Greer Sullivan, JoAnn E Kirchner

**Affiliations:** 1 South Central Mental Illness Research, Education, and Clinical Center (SC MIRECC), Central Arkansas Veterans Healthcare System, 2200 Fort Roots Drive, Building 58, North Little Rock, AR 72114, USA; 2 Center for Mental Healthcare & Outcomes Research (CeMHOR), Central Arkansas Veterans Healthcare System, 2200 Fort Roots Drive, Building 58, North Little Rock, AR 72114, USA; 3 University of Arkansas for Medical Sciences, Psychiatric Research Institute, 4301 W Markham St, #554, Little Rock, AR, 72205, USA

## Abstract

**Background:**

Bottom-up, clinician-conceived and directed clinical intervention research, coupled with collaboration from researcher experts, is conceptually endorsed by the participatory research movement. This report presents the findings of an evaluation of a program in the Veterans Health Administration meant to encourage clinician-driven research by providing resources believed to be critical. The evaluation focused on the extent to which funded projects: maintained integrity to their original proposals; were methodologically rigorous; were characterized by collaboration between partners; and resulted in sustained clinical impact.

**Methods:**

Researchers used quantitative (survey and archival) and qualitative (focus group) data to evaluate the implementation, evaluation, and sustainability of four clinical demonstration projects at four sites. Fourteen research center mentors and seventeen clinician researchers evaluated the level of collaboration using a six-dimensional model of participatory research.

**Results:**

Results yielded mixed findings. Qualitative and quantitative data suggested that although the process was collaborative, clinicians' prior research experience was critical to the quality of the projects. Several challenges were common across sites, including subject recruitment, administrative support and logistics, and subsequent dissemination. Only one intervention achieved lasting clinical effect beyond the active project period. Qualitative analyses identified barriers and facilitators and suggested areas to improve sustainability.

**Conclusions:**

Evaluation results suggest that this participatory research venture was successful in achieving clinician-directed collaboration, but did not produce sustainable interventions due to such implementation problems as lack of resources and administrative support.

## Background

Successfully implementing new clinical practices in real world settings can be very challenging, taking many years for new evidence to reach clinical practice [1-3]. One reason may be that such practices are often implemented in a 'top down' fashion [1-3] -- that is, researchers introduce pre-developed practices or interventions to a clinic or system of care and ask clinicians to assist in implementing them. This approach has been criticized as being ineffective in producing effective translation and sustained implementation of evidence-based practices [4,5]. This purported weakness has been a driving factor behind the increasing emphases for adopting principles critical to the participatory research movement [6,7].

While different models of community-based participatory research (CBPR) have been described in the literature, they usually share a set of common characteristics that emphasize egalitarian collaboration between stakeholders. The belief is that such an approach to research will result in identifying and implementing evidence-based clinical practices more quickly than traditional top-down approaches, and that these changes are more likely to be sustainable over time [8-11]. More often than not, researchers initiate such relationships after securing significant funding from governmental agencies such as the National Institutes of Health. A different approach is to have clinicians with research ideas initiate the relationship with researchers to collaborate on a funded project. Typically, clinicians are unfamiliar with the grant-writing process and require assistance from researchers to help design methodologically sound studies and to attend to such issues as budgeting, human subjects considerations, and project staffing [4,5,12,13]. Nevertheless, this clinician-initiated approach shifts the balance of power toward the clinician. We anticipated that this more active role of clinicians in the technical aspects of the research process would improve sustainability of the interventions by more rapidly building the skills of the clinicians to pursue and secure research funding with less support from researchers.

The Clinical Partnership Program (CPP) at the Veterans Healthcare Administration's (VHA's) South Central Mental Illness Research, Education, and Clinical Center (SC MIRECC), previously described in this journal [14], adopted this approach. Clinicians in four VHA mental health clinics designed and secured funding from the SC MIRECC for four different interventions to improve treatment adherence. We hypothesized that such 'bottom up' projects would have a greater likelihood to be sustained.

Our evaluation of the CPP focused on the following questions:

1. To what extent did each of the projects maintain integrity to its original proposal?

2. To what extent did each of the projects receive a reasonably rigorous evaluation?

3. To what extent was collaboration between clinicians and researchers achieved and what were the facilitators and barriers to collaboration?

4. To what extent were the projects sustained and disseminated over time?

### Community-based participatory research

The advent and development of the CBPR movement over the past several decades [12,13] is based in the writing of Paulo Freire [15] and the work of Kurt Lewin [16]. Many studies have been published illustrating the different methods that have been adopted to successfully implement evidence-based practices in a variety of communities and to sustain them over time [11,17]. The conceptual differences between these methods are less important than the primary goal of addressing the needs of a community with evidence-based treatment paradigms through collaboration of all stakeholders. There have been numerous attempts to outline the critical elements of CBPR, but the most widely recognized conceptualizations emphasize egalitarian participation of all stakeholders, including shared decision-making on all dimensions of a research project, implementing evidence-based interventions, considering the community's strengths and unique characteristics to tailor implementation to result in sustained use of interventions, and developing a long-term relationship of all stakeholders involved. CBPR is better conceived of as an approach to collaboration across multiple research projects where there is no one right method for all issues and all communities on a single research study, but projects do conform to the principles of CBPR over time [12,18,19]. The degree to which key principles of CBPR are incorporated into a given project is largely based upon such factors as funding, feasibility, length of the relationship between partners, and preferences of all stakeholders [20,21].

Multiple layers of partners are common in CBPR for healthcare, including academic researchers, organizational leadership and front-line clinicians, and the patients served by clinicians. Depending upon the nature of the stakeholder relationship, payers (*e.g.*, Medicare, Medicaid, other insurance) may also be a part of the collaborative relationship. Furthermore, the different groups of stakeholders may vary over time. A more limited number of partners may initiate a CBPR project, but then expand to include other perspectives as the logistics of collaboration and trust are built among those who begin to work together. Further, the balance of power and responsibilities is not constant across studies, but changes according to the goals of a given study.

### Setting

The VHA's SC MIRECC collaborates with the network's mental health product line manager and mental health leadership from 10 VHA medical centers and 40 community-based outpatient clinics to promote research, education, and clinical initiatives to improve mental healthcare in the South Central VHA network, which encompasses all or part of eight states in the south central US.

In 2003 the SC MIRECC initiated the CPP [22] to support an empirical exploration of clinical interventions developed by frontline providers, with research and technical support provided by SC MIRECC research mentors. In collaboration with the mental health directors of each of the 10 medical facilities in the network, the broad problem of patient adherence to treatment regimens was designated as the subject of a call for proposals from clinicians. Of the nine program applications received, four were funded. The program lasted 2.5 years and cost approximately $1 million.

The program included clinician-directed projects in Little Rock and Fayetteville Arkansas, New Orleans Louisiana, and Muskogee Oklahoma [22] (Table 1). The clinicians, who varied widely in their prior research experience, were assigned research mentors (statisticians and research design specialists) to coach them through the procedures for conducting Veterans Health Administration (VHA) research, assist with problem solving, and facilitate access to resources. Clinicians were given only as much support as they requested, but regular meetings between mentors and clinical research teams at each site were held to allow mentors to suggest areas to strengthen projects as needed.

**Table 1:** Clinical partnership projects

Project	Project Overview	Design	Project Integrity
1	Investigate whether veterans who used the buddy system (a) improved adherence with medications and medical appointments and/or (b) found increased satisfaction with mental health services.	Quasi-experimental, pre-/post design with a control group N = 39 mental healthcare recipients over three months	Significant design changes; data collection delayed due to administrative requirements; difficulty recruiting subjects

2	Investigate the effectiveness of a brief group intervention to improve combat veterans' engagement in PTSD treatment by increasing awareness about the need to change. The study compared intervention and control groups on process and outcome measures.	Quasi-experimental, pre-/post design with a control group N = 157; 12 month follow-up	No major design changes; data fully collected; Moderate follow-up, with data on all 157 between 3 to 12 months (115 with full 12 months); Project ceased and team members relocated due to Hurricane Katrina.

3	Investigate effect of cognitive behavioral therapy to improve veterans' treatment adherence and improved family involvement in care between veterans randomly assigned to low- and high-reinforcement groups.	Quasi-experimental, pre-/post design with a control group N = 100 over two years	Target n-size reached at baseline, but follow-up incomplete; no major design changes

4	Investigate an intervention that supplemented normal appointment scheduling with additional care to improve mental health patient treatment adherence and reduce no-show rates in a community-based outpatient clinic.	Quasi-experimental, pre-/post design with a control group N = 601 and 208, for two cohorts of mental healthcare recipients	Project was scaled back from initial plan; Most data collected as planned; National VA policy change resulted in contamination between groups

## Methods

To evaluate the program, we viewed each site as a case study in implementation. Quantitative data was collected from surveys and archival data. Qualitative data resulted from separate focus groups of each project's clinicians and mentors. All data were collected within six months following each project's closure. We adapted a model proposed by Naylor [23] to evaluate the collaborative research process across six dimensions (identification of need, research activities, use of resources, evaluation methods, indicators of success, and sustainability) using four different categories, indicating the balance of control exerted by each partner (Table 2).

**Table 2:** Participatory research domains along collaboration scale

	COLLABORATION SCALE			
**Participatory Research Domains**	**Full control by Mentors 1**	**Cooperation 2**	**Participation 3**	**Full control by Clinicians 4**

**Identification of need**	Issues predetermined by mentors, who 'sell' program to clinicians	Clinicians offer advice and input, mentors make decisions	Equal decision making	*Clinicians control decision making, mentors advise*

**Definition of actual research activities**	Issues are predetermined by researchers, who then 'sell' the program to clinicians	Clinicians offer advice and input, but researchers make the decisions	*Equal decision making*	Clinicians control decision making, mentors advise

**Use of resources**	Heavy influx of outside resources	*Outside funding is still the most important but 'in-kind' contributions may be included*	Balanced funding	Small amounts of seed money

**Evaluation Methods**	Tests, surveys, and interviews designed and conducted by mentors with use of hypothesis testing, with significance or results statistically determined	Tests, surveys, and interviews designed by mentors and conducted by the clinical community with use of hypothesis testing, with significance of results statistically determined	*Partnership in design and conduct using multiple methods of data collection in a natural context*	Advice from mentors is sought on design, conducted 100% by the clinicians by using multiple methods in a natural context

**Indicators of success**	Clinicians learn little, and mentors have difficulty sharing power	Clinicians take only marginal responsibility and depend heavily on mentors	Power is shared, but with great tensions	*Clinicians learn new skills, and mentors and clinicians both want to work together*

**Sustainability**	The project dies at completion of the research	*Some spin-offs are produced*	The program continues	The program continues, and new programs are initiated

Note: Italicized text indicates the group consensus ratings of collaboration by clinicians and mentors.

In the original model proposed by Naylor and colleagues [23], community partners and researchers were asked to individually characterize the type and degree of collaboration across the six dimensions using four different categories (as illustrated in Table 2). Focus groups were then held with the partners at each participating site to discuss each person's ratings with the goal of coming to a site-specific consensus rating. The discussion that ensued was the primary focus of analysis. The only known evaluation of this model [20] found the model useful, but noted some ambiguity in the definition of the domains. Thus, for the present evaluation, greater clarity in domain definition was provided to respondents, and the model was supplemented by including additional methods in the evaluation.

### Sample

The CPP program director and SC-MIRECC director, as well as every research mentor and technical assistant (n = 14; 80% female), clinician principal investigator and collaborating investigator (n = 17; 76% female), participated in the evaluation. Clinicians included psychiatrists (n = 4), psychologists (n = 3), nurses (n = 3), social workers/addiction therapists (n = 3), administrative assistants (n = 3), and a research assistant.

Eight mentors were health services researchers, two were methodologists/analysts, and four were administrative coordinators. Two research mentors (*i.e.*, the program director and a coordinator) were involved with every site; thus, there were only eight unique mentors. Participation was voluntary and written informed consent was obtained.

### Data collection

Participating clinicians completed a survey that characterized collaboration across six domains by choosing the degree to which clinicians controlled activities on a scale from 1 (full control by mentors) to 4 (full control by clinicians). A focus group was then held for each site, through which a site-level rating was agreed upon by participants (Table 2). The same process was followed for mentors. Although the focus was on reaching a group consensus of the ratings of collaboration described above, probe questions sought clarification and additional detail about participants' ratings in each domain. These discussions were audiotaped and transcribed verbatim. Clinicians elaborated on these data via email when questions arose during analysis. In addition, the program and research center directors supervising the program participated in face-to-face interviews focusing on: research experience of project personnel; the degree to which projects pursued the proposed plan of research; sites' data collection and analysis; effectiveness of the interventions; intervention sustainability; and dissemination of results (Table 3). Descriptive project information was also collected from archival data (proposals and final reports).

**Table 3:** Project outcomes by site

Project	Familiarity with Research*	Integrity to Proposed Research**	Collected Data	Completed Data Analysis	Intervention Was Effective	Project Was Sustained locally	Results Were Disseminated (Presented)	Results Were Disseminated (published)
1	1	55%	Yes	Yes	No	No	Yes (nationally)	No

2	5	83%	Yes	Yes	No	No	Yes (locally)	No

3	3	85%	Yes	Yes	Yes	No	Yes (locally)	No

4	2	83%	Yes	Yes	Unable to detect	Components adopted	No	No

*Scale = 1 (little research experience) to 5 (much research experience).

**Scale = 0% to 100%

### Data analysis

A chi-square goodness of fit test was used to examine the difference between mentors' and clinician researchers' ratings of collaboration for each site so that ratings could be combined for an overall rating by site. While lack of difference between mentors and clinicians does not mean equivalence, the sample size does not permit for a test of equivalence -- the result of which would be unreliable and uninterpretable. As is often the case with implementation research, sample size does not permit the use of an ideal statistical test. Results of this qualitative data analysis focused on the processes of conducting the projects, their outcomes, and barriers and facilitators to collaboration and project execution. Three evaluation team members experienced in qualitative data analytic techniques independently coded each transcript and subsequently reached consensus through discussion.

## Results

### Degree of project integrity

The degree to which the projects pursued their originally proposed research aims was rated by the research center and program directors on a scale from 0% (no integrity) to 100% (exactly as proposed) (Table 3). Three sites were rated above 80% and one was rated 55%. The project rated lowest for project integrity (project one) was one where the mentor altered original research objectives, and also had the clinician team least experienced in conducting research.

### Degree of methodological rigor

Each study was a clinical demonstration project, employing quasi-experimental, pre/post designs with a control group (Table 1). Studies used convenience samples, with participants randomly assigned to a control or intervention group. Sample sizes ranged from 39 to over 200. Only Project 2 achieved a sample as originally proposed. Two projects required reductions from their projected sizes, Project 1 due to difficulty in recruiting and Project 4 due to the elimination of one intervention group. Project 3 recruited participants as planned at baseline, but had significant difficulty in retaining the sample for follow-up.

Although all projects completed sufficient analyses to report final outcomes, data management and analyses posed significant difficulties. The clinicians relied almost entirely upon research center statisticians for data management and analyses, and in the qualitative interviews, all clinical site personnel noted delays when consultants lacked sufficient time to analyze project data.

Project 4 suffered potential dilution of intervention effects due to the issuance of a national VA performance measure halfway through the project that mandated clinical procedures that included the key intervention being tested. Project 2 lost access to all of its data during analyses due to destruction of its facility from Hurricane Katrina. Thus, methodologically, projects most commonly confronted difficulties with subject recruitment, data management, and data analyses.

### Dissemination

Three projects presented results at scientific meetings (two local and one national). All projects completed final reports, but none resulted in a published manuscript. In the focus groups, three sites expressed the desire to publish from their experiences, but cited time as a barrier to writing. The intention of the CPP model was that clinicians assume this role, with the research consultants assisting only upon request.

### Degree of collaboration

There was substantial agreement between the clinicians' and mentors' perceived levels of collaboration across our six domains (Table 4). Sample size precluded the ability to reliably test equivalence of the ratings. However, a chi-square goodness of fit test between the two sets of ratings yielded non-significant results (χ^2 ^= 4.67, df = 23, n.s.), indicating that the distributions of ratings did not significantly differ between groups. No domain rating differed between groups by more than a single point, and there was no consistent pattern in the minor rating differences (*e.g.*, one group consistently rating domains as more collaborative than the other group). Thus, the ratings were averaged for each domain. Note that no category of collaboration is inherently better or worse than another. Category assignment is purely descriptive.

**Table 4:** Collaboration ratings by site

Site	Group	Identification of Need	Definition of Research Activities	Use of Resources	Evaluation Methods	Indicators of Success	Sustainability
1	Clinicians	4	3	3	3	4	4
	
	Mentors	4	4	2	3	4	3

2	Clinicians	4	3	2	3	4	2
	
	Mentors	4	3	1	3	4	1

3	Clinicians	3	3	3	4	4	4
	
	Mentors	4	3	2	4	4	4

4	Clinicians	3	4	2	3	3	2
	
	Mentors	4	4	2	3	4	3

	Mean (SD)	3.75 (0.46)	3.38 (0.52)	2.13 (0.64)	3.25 (0.46)	3.88 (0.35)	2.88 (1.13)

Note: Collaboration Scale = 1 (full control by mentors), 2 (cooperation), 3 (participation), 4 (full control by clinicians)

In general, both clinicians and mentors characterized the collaborative process as weighted towards full control by the clinicians in two domains: 'identification of need' (mean = 3.75, SD = 0.46) and 'indicators of success' (mean = 3.88, SD = 0.35). Participants viewed 'definition of research activities' (mean = 3.38, SD = 0.35) 'evaluation methods' (mean = 3.25, SD = 0.46), and 'sustainability' (mean = 2.88, SD = 1.13) as having occurred through equal decision making. 'Use of resources' was rated at the level of cooperation (mean = 2.13, SD = 0.64).

### Facilitators and barriers to collaboration

#### Clinicians conducting research

Clinicians identified the program's provision of funding and technical assistance as a key strength because it enabled them to pursue research that would not otherwise have been likely. Clinicians particularly valued the mentors' methodological expertise and their knowledge of and assistance negotiating the VHA's organizational complexities -- especially when hiring personnel and securing human subject approvals.

There was a wide range of experience across the clinical sites in terms of clinicians' prior experience and knowledge of research. On a scale from 1 (little research experience) to 5 (much research experience), the program and research center directors rated experience as a 1, 5, 3, and 2 (Table 3). Clinicians at some sites expressed dissatisfaction with the statistician consultants because there were often time lags in communication. Mentors noted their initial underestimation of sites' preparedness to negotiate research logistics, stating that the clinical sites seemed unaware of the extent and time-consuming nature of the activities the mentors performed on their behalf.

Research mentors noted problems securing clinical leadership support at all sites, especially inadequate relief from clinical duties for the clinicians. Consequently, clinicians often donated research time while maintaining a full clinical workload, or had to significantly limit their involvement in research. Mentors reported occasionally intervening on behalf of the clinicians to remind clinical leaders of the time requirements of research and intended use of funds. These issues were perceived to affect the overall success of the projects and willingness of clinicians to engage in future research. Both groups suggested that local administrators be involved in the planning stages of future clinician-directed projects to assure tangible commitments.

### Mentorship/communication

Respondents reported that the collaboration enhanced rapport between the research center and the clinical sites. With few exceptions, clinicians noted that they felt supported and enjoyed working with research center personnel, whom they described as being as professional and helpful as possible. Three of the four sites expressed that they had become more confident and capable of conducting research in the future. Likewise, mentors reported that they developed a better understanding of the clinical sites.

Research mentor turnover was a significant difficulty, with all sites experiencing at least one change in their primary mentor. All focus groups mentioned these changes as having resulted in disruptive shifts in established relationships, a feeling of instability for clinicians, and time lapses for new mentors to be assigned and be oriented to the project.

Although the clinicians reported that mentors were available for assistance, they cited such communication barriers as the mentor using too much research jargon, which led to confusion and wasted time and effort. In addition, one clinical site initially perceived the research mentor's role as micromanaging the research or providing punitive oversight -- an impression that only changed after a long-term working relationship developed.

Clinicians stated that at times their perspectives differed from those of the mentors in terms of overall project goals, leading to some frustration. One group of clinicians highlighted the differences in the mentors' level of research expertise, noting that clinicians preferred mentors who could increase the clinicians' research skills, as opposed to providing only administrative assistance.

### Degree of sustainability

Respondents defined sustainability in two ways: sustainability of the interventions and sustainability of the relationship between the clinicians and research center. Clinicians were largely open to future collaborations with the research center, even when their interventions did not continue (see Table 3). Thus, participants rated the projects at the cooperation level ('some spin-offs produced,' see Table 2). Three stopped altogether, but one saw several core elements adopted clinically following the project period (Project 4). Of those that stopped, Project 2 ceased with the destruction of the VA hospital and relocation of all project personnel after Hurricane Katrina. Respondents attributed Projects 1 and 3 terminations to the lack of support from clinical staff and facility leadership. 'Indeterminate research results' (Project 3) was also cited as a factor.

## Discussion

The purpose of the CPP was to equip clinicians with the necessary resources to develop, implement, and evaluate their own ideas for clinical innovations in mental healthcare. It was anticipated that this 'bottom-up' approach would increase the likelihood of the interventions being sustained if they were found to be effective. One concern with clinician-directed, as opposed to researcher-directed, programs is the possibility that the resulting empirical evidence would be less valid and reliable due to less rigorous project implementation. Thus, we used several indices to evaluate how well the interventions were executed and their contributions to the field.

Our evaluation suggests that the program was effective in several respects, but key outcomes (*i.e.*, sustainability) were not realized.

### Program challenges

Community relevance and sustainability of clinical programs are arguably two of the most important criteria for successful implementation of clinical interventions. Relevance refers to the applicability of a particular program in clinical settings that originates from the mutual input of community members (*e.g.*, front-line clinicians) and researchers. Sustainability not only refers to the continuation of an evidence-based intervention that begins through a single research study, but also the collaborative relationship built between researchers and a community across projects. Sustainability is a key dimension of any participatory project because this has been shown to be one of the most pervasive weaknesses of top-down research endeavors [12], often because the needs of the community are not addressed [24]. While relevance was carefully considered by involving the mental health directors in defining the call for proposals and by letting the clinicians develop their own interventions to test, sustainability was not realized in most of the sites. Collaboration clearly impacted the projects' implementation, but it is less clear whether continuing collaboration could have increased the sustainability of the interventions over time [24]. One of the anticipated benefits of the model described in this report assumed that the allocation of more power to the clinicians might make the tested program more sustainable than in the other three models of research with communities. Most projects noted mutable characteristics of their sites (*e.g.*, increased support from clinical leadership) that may have impeded the continuance of interventions. The importance of such organizational factors was clearly seen for the site impacted by the performance measure initiative. This site continued and extended its intervention when the work of the clinicians was bolstered by clinical practice mandates.

Some difficulties arose from mentor communication and turnover. It became clear in retrospect that a more effective model of mentoring would be to ensure a good match between mentors and sites, along with programmatic mechanisms to minimize the disruption to the relationship if a mentor were to change positions.

A significant underestimation in the program concerned the resources that would be necessary to assist the clinician teams to conduct well-designed demonstration projects. Personnel costs alone were enormous, not to mention the additional uncompensated time donated by mentors and clinicians to conduct the projects. This latter fact may have been a primary cause for some mentor turnover.

It is important to note the similarity of the challenges that arose in the project to those with traditional top-down research. Seasoned researchers anticipate difficulties with subject recruitment, administrative support, and adoption of programs by peers. Mentorship provided the intellectual resources (*e.g.*, experience) to work through these issues, but those efforts resulted in equalizing the clinicians to experienced researchers in terms of solving common challenges in conducting research. Thus, allocating extensive resources into a different approach did not eliminate challenges inherent in research; it just equipped the clinicians to deal with them and did not result in a greater likelihood of the interventions being sustained.

Disseminating the finds of a project to the participating communities (site clinicians, leadership, and patients) and to the larger scientific community is an important element of CBPR. However, this was only partially realized in this program. Mental health leadership was informed of the results of the projects and evaluation of the collaboration between clinicians and mentors, but publications and conference presentations were not common. Dissemination may have been enhanced by assigning greater leadership responsibilities to the research mentors who are most experienced in preparing conference presentations and publications. Additionally, as is often a weakness in CBPR, patients were not systematically involved in the development of the interventions or dissemination of the results [21,25-28], which may have enhanced both the relevance of the projects and potentially their sustainability.

### Program strengths

Most effective was the degree to which mentors and clinicians collaborated across the projects. This relationship was critical to the execution of the projects, where technical assistance, access to resources, and encouragement allowed each to progress to completion. Several difficulties and misunderstandings were noted, but all participants expressed an overall positive review of their interactions. In fact, although clinicians did not have full control over every aspect of the collaboration, this was never intended. This is the precise reason why mentoring teams were established to assist with the funding and methodological components of the grants. Even under this model, the clinicians indicated that they had learned a great deal about research and would be more prepared for greater independence in a future project.

The CPP would ideally have the capacity-building potential to increase the likelihood of clinicians conducting additional research in the future. While this was not evaluated in depth, long-term follow up of such programs would be instructive. It is not clear whether providing intensive support in the technical aspects of conducting research handicaps clinicians from obtaining future grants that do not have a mentoring component to them. We do not know objectively, beyond the clinicians' self report, to what extent clinicians developed the skills necessary to be more independent in subsequent research projects.

### Looking ahead

A growing body of research (including that in the same settings within which these projects took place) [14] has shown that sustainability is an implementation issue, not necessarily achievable by bottom-up clinical demonstration projects alone. Implementation techniques need to be incorporated into any demonstration project for it to continue beyond the funding period. Site readiness to change, tangible administrative support and commitment, and clinician experience in research seem critical to both sustainability and the cost of a bottom-up partnership program.

Given the barriers to conducting the research projects and sustaining them past the funding period, the skills of mentors should be carefully considered. In this project, mentors were often called upon to serve as facilitators with mental health leadership and with other site-level organizational characteristics for project implementation. Thus, this suggests that the skills and roles of mentors must not only involve the technical aspects of conducting research, but also those involved in making lasting clinical changes in a system of care -- *i.e.*, they should be well versed in implementation techniques and community engagement [27]. In this manner, mentors serve as facilitators of the projects and sustainability. Minimizing the project management demands of mentors by recruiting less research naïve clinicians and assisting clinicians to hire strong project staff would allow mentors to take on this different role.

Practice-based research networks (PBRNs) are interesting relationships of healthcare stakeholders for the purposes of training and research that may or may not conform to the ideals of CBPR [11,26], but are an ideal organizational structure to pursue participatory research. It has been estimated that there are over 100 PBRNs in the US [27,29], and the intent of the majority is to foster fully cooperative research relationships between communities and academic institutions. Clinics in the networks are ideally intimately involved with the generation of research as researchers, rather than only serving as locations where research can be conducted. This approach is similar the one described in the manuscript; however, more control and responsibility were given to clinicians with the intent that the experience could lead to greater research capacity building in the clinics and hence, a more rapid move toward future clinician-initiated research. This assumption seemed only partially correct, as there was still a very heavy reliance upon the research mentors, just as would be the case if researchers had initiated the projects.

Several features of our evaluation limit generalizability. First, the sample was small. However, considering the diversity across sites, the results were not only informative, but establish a baseline measure of this type of program. Second, the six-month delay between the conclusion of the programs and focus group evaluations raises concerns about recall accuracy; however, the focus group format allowed participants to refresh each other's memories. Lastly, the data used for this evaluation are descriptive in nature and cannot lead to firm predictions about what can and cannot lead to intervention sustainability.

### Summary

Program evaluation yielded mixed findings. While clinicians and research mentors reported that collaboration improved relationships and produced research that would not otherwise have been possible, sustainability and the academic impact of the research conducted was minimal. There was a degree of naiveté in the assumption that simply providing resources and support would increase sustainability of the clinical interventions. Future similar programs should carefully consider clinicians' prior research experience, ensure a stable mentoring environment, and prioritize sites that have a readiness to change and committed administrative support for research.

## Competing interests

The authors declare that they have no competing interests.

## Authors' contributions

DB oversaw the design, data collection, analysis, and led the preparation of this manuscript. MSF participated in data collection and analysis. CE was involved with the interpretation and presentation of these results, and the editing and submission of this manuscript. GS led the conceptual development of this program as Director of the funding center and provided data described below. JAK oversaw the Clinical Partnership Program and the conceptual design of the evaluation, and provided data described below. All authors have read and approved the final manuscript.
